# The chloroplast genome of the desiccation-tolerant moss *Pseudocrossidium replicatum* (Taylor) R.H. Zander

**DOI:** 10.1590/1678-4685-GMB-2018-0184

**Published:** 2019-07-18

**Authors:** Miguel A. Cevallos, Gabriela Guerrero, Selma Ríos, Analilia Arroyo, Miguel Angel Villalobos, Helena Porta

**Affiliations:** 1 Centro de Ciencias Genómicas, Programa de Genómica Evolutiva, Universidad Nacional Autónoma de México, Cuernavaca, Morelos, Mexico; 2 Centro de Investigación en Biotecnología Aplicada, Laboratorio de Biología Molecular y Biotecnología de Plantas, Instituto Politécnico Nacional. Tepetitla de Lardizabal, Tlaxcala, Mexico; 3 Instituto de Biotecnología, Departamento de Biología Molecular de Plantas, Universidad Nacional Autónoma de México, Cuernavaca, Morelos, Mexico

**Keywords:** Bryophytes, Pottiaceae, chloroplast, next-generation sequencing

## Abstract

Mosses in conjunction with hornworts and liverworts are collectively referred to as bryophytes. These seedless, nonvascular plants are the closest extant relatives of early terrestrial plants and their study is essential to understand the evolutionary first steps of land plants. Here we report the complete chloroplast (cp) genome sequence of *Pseudocrossidium replicatum,* a moss belonging to the Pottiaceae family that is common in the central highlands of Mexico, in South America, in southern USA, and in Kenia. The cp genome (plastome) of *P. replicatum* is 123,512 bp in size, comprising inverted repeats of 9,886 bp and single-copy regions of 85,146 bp (LSC) and 18,594 bp (SSC). The plastome encodes 82 different proteins, 31 different tRNAs, and 4 different rRNAs. Phylogenetic analysis using 16 cp protein-coding genes demonstrated that *P. replicatum* is closely related to *Syntrichia ruralis,* and the most basal mosses are *Takakia lepidozioides* followed by *Sphagnum palustre*. Our analysis indicates that during the evolution of the mosses’ plastome, eight genes were lost. The complete plastome sequence reported here can be useful in evolutionary and population genetics.

The chloroplast (cp) is not only the organelle where photosynthesis takes place, but also the site where a wide variety of functions essential for plant metabolism occur, like nitrate and sulfate assimilation, synthesis of almost all amino acids and fatty acids, chlorophylls, and carotenoids (Jensen and Leister, 2014). The cp genome (plastome) consists of a single circular double stranded DNA molecule, and for most of the land plants its structure is well conserved. The plastome can be divided into four regions: two copies of an inverted repeat (IR) region, a large-single-copy (LSC) region, and a small-single-copy (SSC) region. This pattern is maintained through a flip-flop homologous recombination mechanism (Palmer *et al.*, 1983). Gene number and content of plastomes is highly conserved, however, the sequence of the non-coding intergenic spacer regions varies to a large extent. In several plant lineages, comparative genomics studies have shown structural changes that include gene or intron losses, loss of an IR region, inversions, and other kinds of genome rearrangements (Yurina *et al.*, 2017). Considering these features, together with the cp uniparental mode of inheritance and that their genomes are almost recombination free, the study of cp genome sequences is an invaluable tool to resolve taxonomic issues and to understand the origin and evolution of the plant kingdom.

Bryophytes are a paraphyletic group of land plants that comprises hornworts, liverworts, and mosses. A common characteristic of this heterogeneous group is that its sporophyte (diploid phase) is unbranched and contains a single sporangium that is attached to the gametophyte, the photosynthetic and haploid phase of the bryophyte life cycle. Bryophytes are the most primitive terrestrial group of plants that have recently emerged as models for plant biology studies. Particularly mosses have been receiving more attention because their gametophyte is haploid, they display low anatomical complexity, and have a rapid cell cycle (Cove, 2005). Mosses are a highly diverse clade containing more than 12,800 species (Delgadillo-Moya, 2014). The growth and sexual reproduction of these land plants is limited to the sites were water is available, however, they posses mechanisms that allow their survival during the dry season. Today, around 1750 complete plastomes of land plants have been determined (NCBI, 2016): 15 correspond to Bryophyte cp, and of these only nine belong to mosses (Rensing *et al.*, 2009). To have better understanding about the origin and evolution of the first land plants many more cp sequences are needed.

*Pseudocrossidium replicatum,* belongs to the moss family Pottiaceae and can be found in the central highlands of Mexico, South America, southern USA, and Kenia. In the work presented here and with the aim to expand our understanding of these primitive land plants, we present the complete genome sequence of the *P. replicatum* plastome and the comparison of its structure with the other moss cp genomes.

Our *P. replicatum* specimen (sporophyte and gametophyte) was collected in Ixtacuixtla, Tlaxcala, Mexico (19-20-03.3 N, 98-21-59.9 W, 2159 msnm), in January 2008. An *in vitro* culture was started from a single spore and maintained since then through weekly subcultures in a growth chamber at 23 ºC and 30% of relative humidity, with a photoperiod of 16/8 h light/dark. The genomic DNA used in this study was extracted from 7 day-old protonemata, cultivated *in vitro* in PpNH4 culture medium, using the *ZR Plant/Seed DNA Microprep* kit and following the manufacturer’s instructions (ZymoResearch). A DNA pair-end library (PE 2 x 75) for Illumina NextSeq500 platform was prepared and sequenced at *Unidad Universitaria de Secuenciación Masiva de DNA de la Universidad Nacional Autónoma de México*. Reads were assembled with SPAdes-3.9.0 and all contigs that matched the cp genome of *Syntrichia ruralis* were used to orient *P. replicatum* contigs (Bankevich *et al.*, 2012). We selected a minimal contigs set that covered all the *S. ruralis* plastome. Overlapping regions between contigs and borders between the inverted repeat regions were confirmed by PCR and Sanger sequencing. The genome was annotated with the Dual Organellar GenoMe Annotator (DOGMA), BLAST homology search, and tRNAscan-SE (Wyman *et al.*, 2004; Lowe and Chan 2016). The circular genome map was obtained with the Organellar Genome DRAW (Lohse *et al.*, 2013). Protein alignments were done with Clustal_Omega, and a Neighbor joining phylogenetic tree was constructed with MEGA7, using the concatenated alignment of 16 protein sequences (Sievers and Higgins, 2014; Kumar *et al.*, 2016). The complete cp genome of *P. replicatum* with its gene annotation was deposited in NCBI GenBank with accession number MG132071.

The complete cp genome of *P. replicatum* has a total length of 123,512 bp. It possesses two inverted repeats (IRs), each one of 9,886 bp, a large single copy (LSC) region of 85,146 bp, and a small single copy (SSC) region of 18,594 bp (Figure 1). The cp genome of this organism posses 125 genes, with 82 encoding different proteins, 31 different tRNAs, and four different rRNAs. Among the protein encoding genes, most are involved in photosynthesis functions, including genes encoding the photosystems I and II, products involved in the assembly and stability of photosystem I, the large subunit of Rubisco, cytochrome b6/f complex, cytochrome c synthesis, and the eleven subunits of the NADH-plastoquinone oxidoreductase. Genes encoding the six subunits of the ATP synthase are also present. Additionally, this genome encodes proteins participating in transcription and translation, like the subunits of RNA polymerase, 21 ribosomal proteins, the translational initiation factor 1, and MatK, an intron maturase (Table 1).

**Figure 1:**
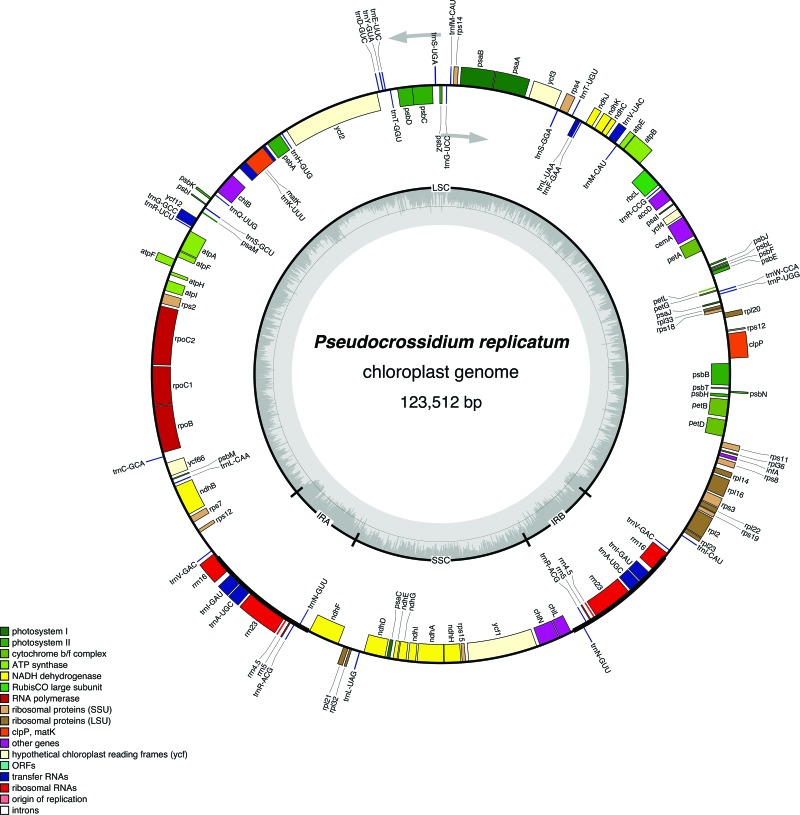
Gene map of the moss *P. replicatum*. Genes in the inside of the map are transcribed clockwise, and genes in the outside of the map are transcribed counterclockwise. Exons are indicated as closed boxes. Genes with related function are shown in the same color.

**Table 1:** Genes encoded in *Pseudocrossidium replicatum* cpDNA.

Gene categories	Group of genes	Gene names
Photosynthesis	Rubisco	*rbcL*
	Photosystem I	*psaA, psaB, psaC, psaI, psaJ, psaM*
	Assembly/stability of photosytem I	*ycf1, ycf2,* *ycf3*, *ycf4, ycf12,* *ycf66*
	Photosystem II	*psbA, psbB, psbC, psbD, psbE, psbF, psbH, psbI, psbJ, psbK, psbL, psbM, psbN, psbT, psbZ*
	ATP Synthase	*atpA, atpB, atpE, atpF, atpH, atpI*
	Cytochrome b/f complex	*petA,* *petB* *,* *petD*, *petG, petL*
	Chlorophyll biosynthesis	*chlB, chlL, chlN*
	NADPH dehydrogenase	*ndhA*, *ndhB*, *ndhC, ndhD, ndhE, ndhF, ndhG, ndhH, ndhI, ndhJ, ndhK*
Transcription and translation	Transcription	*rpoB,* *rpoC1*, *rpoC2*
	Ribosomal proteins	*rpl2*, *rpl14,* *rpl16*, *rpl20, rpl21, rpl22, rpl23, rpl32, rpl33, rpl36, rps2, rps3, rps4, rps7, rps8, rps11, rps12, rps14, rps15, rps18, rps19*
	Translation initiation factor	*infA*
RNA	Ribosomal RNA	*rrn5, rrn16, rrn4.5, rrn23S,*
	Transfer RNA	*trnA-UGC*, *trnC-GCA, trnD-GUC, trnE-UUC, trnF-GAA,* *trnG-GCC*, *trnG-UCC, trnH-GUG, trnI-CAU,* *trnI-GAU*, *trnK-UUU*, *trnL-CAA,* *trnL-UAA*, *trnL-UAG, trnM-CAU, trnN-GUU, trnP-UGG, trnQ-UUG, trnR-ACG, trnR-CCG, trnR-UCU, trnS-GCU, trnS-GGA, trnS-UGA, trnT-GGU, trnT-UGU, trnV-GAC,* *trnV-UAC*, *trnW-CCA, trnY-GUA, trnfM-CAU*
Other	RNA processing	*matK*
	Carbon metabolism	*cemA*
	Proteolysis	*clpP*
	Other proteins	*accD*

Underlined genes contain introns.

The two inverted repeat regions contain the rRNA genes (23S, 16S, 5S and 4.5S) and five tRNA genes, each. Within the SSC region lay seven of the genes encoding the NADH-plastoquinone oxidoreductase along with three genes encoding ribosomal proteins and four genes involved in other functions (*chlL*, *chlN*, *ycf1* and *psaC*). This organization remains fairly constant in the moss plastomes compared here. The rest of the genes are located in the LSC region.

Twelve protein-encoding genes and six different tRNA genes have introns. The largest one, with 2,259 bp, belongs to the *trnK-UUU* genes and is large enough to embrace the complete *matK* gene. Additionally, two genes, *atpF* and *rpoB,* are possibly transcribed and modified by RNA editing.

Until now and including the plastome reported here, there are 10 sequenced moss cp genomes: *Nyholmiella obtusifolia* (NCBI: NC_026979.1), *Orthotrichum rogeri* (NCBI: NC_026212.1)*, Syntrichia ruralis* (Oliver *et al.*, 2010), *Physcomitrella patens* (Sugiura *et al.*, 2003)*, Sanionia uncinata* (Park *et al.*, 2018)*, Sphagnum palustre* (Shaw *et al.*, 2016)*, Takakia lepidozioides* (NC_028738) *Tetraphis pellucida* (Bell *et al.*, 2014), and *Tetraplodon fuegianus* (Lewis *et al.*, 2016). These 10 species are representatives of eight different moss families. These plastomes vary in size, gene content and, to a lesser extent, in the location of the genes in the LSC/IRB/SSC/IRA boundary regions.

The length of the *P. replicatum* cp genome (123,512 bp) falls within the range reported for the other moss plastomes, considering that *S. rularis* has the smallest plastome (122,630 bp) and *T. lepidozioides* the largest (149,016 bp). The total GC content of the *P. replicatum* cp genome is 28.2%, which is similar to that of its closest relative (28.38%). In general, we found that the moss cp genomes have a very similar repertoire of genes, though we were able to identify a few differences in some of them. The largest genomes contain more genes, especially those encoding for proteins: *T. lepidozioides* encodes 91 different genes and *S. palustre* (plastome size of 140,040 bp) encodes 85 distinct proteins. Congruently, the number of protein encoding genes of the *P. replicatum* plastome lies in the average: 82. The number of rRNA genes is the same in all moss plastomes (8 genes). With only one exception, *N. obtusifolia* that contains 32 different tRNAs, the rest encode 31.

To determine the relationships between the moss genomes analyzed here, we constructed a Neighbor joining phylogenetic tree using a concatenated alignment of 16 proteins and including the fern *Osmundastrum cinnamomeum* as outgroup (Figure 2). This figure shows that the most basal chloroplast moss is *T. lepidozioides,* which has the largest plastome and encodes the largest gene set. The next basal cp genome corresponds to *S. palustre* that possesses the second largest plastome and also encodes the second largest gene set. The *T. lepidozioides* plastome possess eight genes that are absent in the other moss cp genomes (*cysA, petN, rps12, rps16, cysT, rpoA ccsA and tufA*), and these probably were transferred to the nucleus in very ancient events. Surprisingly, the *petN* gene is present in *T. lepidozioides* and *P. patens,* suggesting that this gene was lost early in moss evolution, but was regained later by an unknown mechanism in the ancestors of *P. patens*.

**Figure 2:**
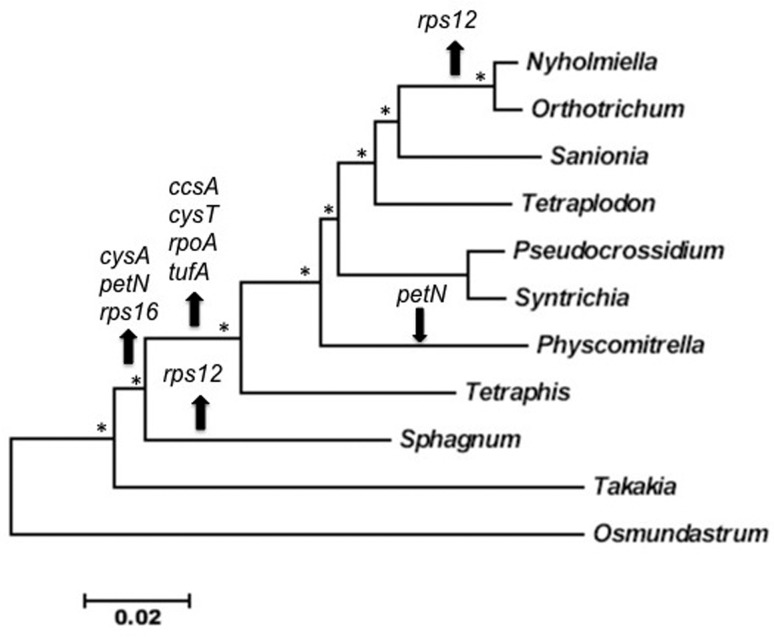
Phylogenetic tree of chloroplast genomes of mosses. A Neighbor joining tree was constructed using a concatenated alignment of 16 proteins: *accD*, *atpA*, *atpB*, *cemA*, *chlB*, *chlL*, *clpP*, *ndhB*, *ndhF*, *ndhH*, *ndhK*, *petA*, *psaA*, *psbC*, *rbcL*, and*rps2*. The chloroplast sequence of the fern *Osmundastrum cinnamomeum* was used as outgroup. Asterisks indicate boot-strap values > 85%. Arrows indicate gene insertion (down) or deletion (up). The scale represents number of amino acid substitution per site.

We found three different patterns in the LSC/IRB/SSC/IRA boundary regions in the moss cp genomes (Figure 3). The first pattern is shared between *T. pellucida, N. obtusifolia, O. rogeri, S. palustre, T. fuegianus,* and *P. replicatum.* The second is represented in the *T. lepidozioides, P. patens, and S. uncinata* plastomes, and the third pattern pertains only to *S. ruralis.* These patterns are not congruent with the phylogenetic tree, indicating that the LSC/IRB/SSC/IRA boundary regions are constantly changing as a consequence of the flip-flop recombination described earlier.

**Figure 3:**
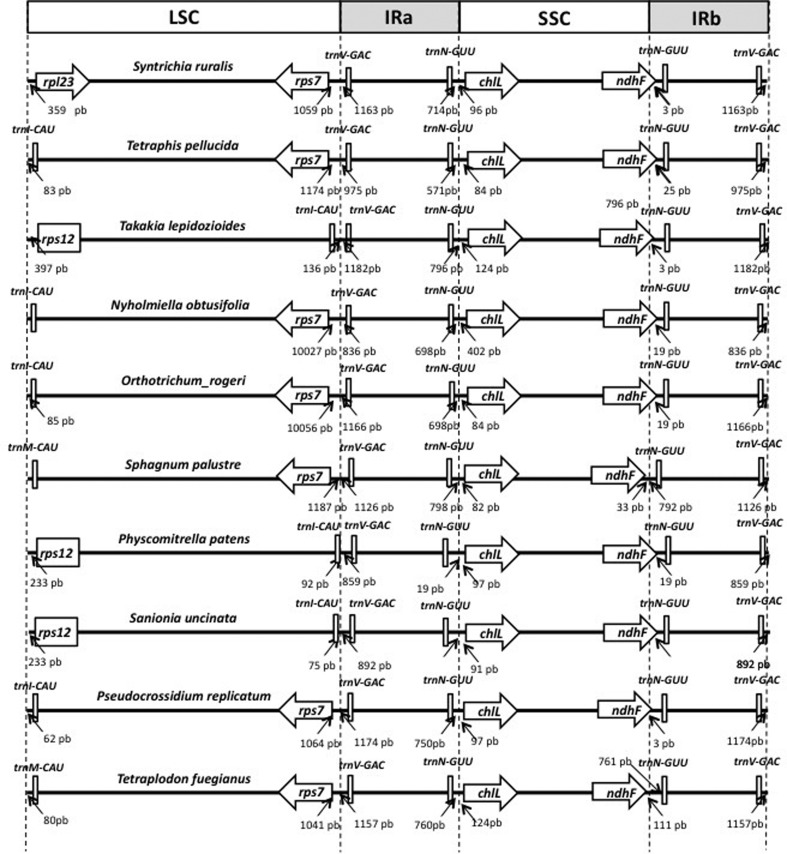
Comparison of chloroplast genome structure between mosses. Shown are the genome distances and gene position in the vicinity of LSC, IRB, SSC, and IRA in the genome structure of mosses cp.
